# Dealing with PET radiometabolites

**DOI:** 10.1186/s13550-020-00692-4

**Published:** 2020-09-23

**Authors:** Krishna Kanta Ghosh, Parasuraman Padmanabhan, Chang-Tong Yang, Sachin Mishra, Christer Halldin, Balázs Gulyás

**Affiliations:** 1 Lee Kong Chian School of Medicine, Nanyang Technological University Singapore, 59 Nanyang Drive, Singapore, 636921 Singapore; 2 Department of Nuclear Medicine and Molecular Imaging, Radiological Sciences Division, Singapore General Hospital, Outram Road, Singapore, 169608 Singapore; 3 Duke-NUS Medical School, 8 College Road, Singapore, 169857 Singapore; 4 Department of Clinical Neuroscience, Center for Psychiatry Research, Karolinska Institutet and Stockholm County Council, SE-171 76 Stockholm, Sweden

**Keywords:** Radiometabolites, PET, HPLC, Radiotracer, HPLC-MS

## Abstract

**Abstract:**

Positron emission tomography (PET) offers the study of biochemical, physiological, and pharmacological functions at a cellular and molecular level. The performance of a PET study mostly depends on the used radiotracer of interest. However, the development of a novel PET tracer is very difficult, as it is required to fulfill a lot of important criteria. PET radiotracers usually encounter different chemical modifications including redox reaction, hydrolysis, decarboxylation, and various conjugation processes within living organisms. Due to this biotransformation, different chemical entities are produced, and the amount of the parent radiotracer is declined. Consequently, the signal measured by the PET scanner indicates the entire amount of radioactivity deposited in the tissue; however, it does not offer any indication about the chemical disposition of the parent radiotracer itself. From a radiopharmaceutical perspective, it is necessary to quantify the parent radiotracer’s fraction present in the tissue. Hence, the identification of radiometabolites of the radiotracers is vital for PET imaging. There are mainly two reasons for the chemical identification of PET radiometabolites: firstly, to determine the amount of parent radiotracers in plasma, and secondly, to rule out (if a radiometabolite enters the brain) or correct any radiometabolite accumulation in peripheral tissue. Besides, radiometabolite formations of the tracer might be of concern for the PET study, as the radiometabolic products may display considerably contrasting distribution patterns inside the body when compared with the radiotracer itself. Therefore, necessary information is needed about these biochemical transformations to understand the distribution of radioactivity throughout the body. Various published review articles on PET radiometabolites mainly focus on the sample preparation techniques and recently available technology to improve the radiometabolite analysis process. This article essentially summarizes the chemical and structural identity of the radiometabolites of various radiotracers including [^11^C]PBB3, [^11^C]flumazenil, [^18^F]FEPE2I, [^11^C]PBR28, [^11^C]MADAM, and (+)[^18^F]flubatine. Besides, the importance of radiometabolite analysis in PET imaging is also briefly summarized. Moreover, this review also highlights how a slight chemical modification could reduce the formation of radiometabolites, which could interfere with the results of PET imaging.

**Graphical abstract:**

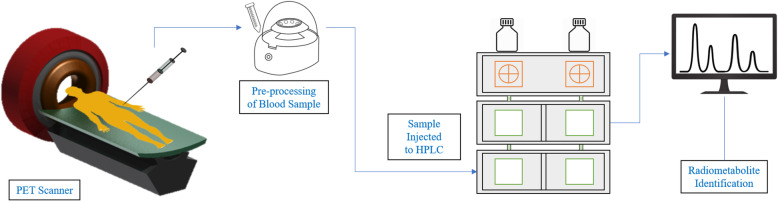

## Background

Positron emission tomography (PET) is one of the most rapidly progressing imaging modalities, which offers understanding about the biochemical, physiological, and pharmacological functions at a molecular and cellular level [1, 2]. To date, PET has been applied in several areas including cardiology, neurology, and oncology particularly in tumor targeting and diagnosis [3, 4]. Numerous PET radiotracers have been studied and developed for imaging cancerous lesions, brain receptors, transporters, and enzyme systems, but only a few of them are currently in use as imaging agents in clinical practice. The PET imaging technique includes the acquirement of physiological images that constitute the distribution of radioactivity associated with positron-emitting radionuclides in the volume of examination [5]. Compared to other imaging tools like the X-ray, magnetic resonance imaging (MRI), and computed tomography (CT) which offer structural information, PET imaging is advantageous as it shows numerous functional and biochemical characteristics of organs and tissues of interest. PET combines several crucial features like absolute quantitation, allows repetitive and longitudinal examination before/after interventions with the help of short-lived PET radionuclides. Very high resolution (< 2.5 mm) can be achieved at any part of the body with a dedicated PET scanner [6, 7]. Most importantly, procedures developed in preclinical research can be applied to clinical studies. The PET imaging technique is currently the only non-invasive imaging tool to measure in vivo tissue pharmacokinetics quantitatively.

The process involved in developing new PET radiopharmaceuticals begins with compound selection, through in vitro and in vivo efficacy evaluation, ultimately leading to clinical investigation. Hence, the performance of a PET study mostly depends on the used radiotracer of interest. However, developing PET imaging tracers is difficult as various critical criteria need to be fulfilled such as optimum molecular weight and lipophilicity. Besides, the radiotracer should have a high affinity for the target receptor, and it should show a fast washout after reaching the saturation binding. For imaging the central nervous system (CNS), the radiotracer should not be a subject for P-glycoprotein (P-gp) [8–10]. On the other hand, the non-brain tissue radiotracer development process also faces some serious challenges as low peripheral radiometabolism is necessary to compensate for the lack of BBB. In the case of the CNS radiotracer development, most of the compounds failed to show promising results in preclinical and clinical studies. Some of the most common causes for the failure of radiotracers appear when non-displaceable binding surpasses target binding, i.e., when signals from the background exceed the signals coming from the target. One of the serious problems with PET is that it offers information about the total radioactivity deposited in the tissue of interest but does not reflect any evidence of its chemical identity. The existence of radiometabolites in the tissue of interest would affect how precise the quantitative analysis of the PET examination is [11, 12].

In living organisms, PET radiotracers usually encounter different types of chemical modifications including oxidation, reduction, hydrolysis, decarboxylation, and various conjugation processes. Due to these biotransformations, diverse chemical entities are generated while the concentration of the original radiotracer decreases [13–18]. Therefore, the signal measured by the PET scanner replicates the entire amount of radioactivity deposited in the tissue of choice, but it does not offer any indication about the chemical identity of the pharmacophore itself. From a radiopharmaceutical perspective, the quantification of only the parent radiotracer fraction present in tissues is a requirement [19]. Almost all the PET radiotracers are metabolized within the body. Among these metabolized products, some may carry the radioactivity tag. There are some reactions that are planned, such as the phosphorylation of [^18^F]FDG and its resulting trapping into the cell. However, the PET radiotracers are mostly metabolized to form unwanted radiometabolites which create serious problems in quantification. Although radiometabolites present in plasma can be rectified, the radiometabolites deposited into tissues should be considered during image analysis. Radiotracers for brain imaging are designed in such a way that they do not produce any radiometabolites that can pass through the blood–brain barrier [20]. However, there are some exemptions to this guideline, as in the case of [^18^F]FDOPA. In the preclinical evaluation of a new PET radiotracer, it is important to take into consideration that the radiometabolites may display a significant difference in distribution patterns and activity in the body in comparison to the parent radiotracer. Enough information on biochemical transformations is crucial to have a more detailed knowledge of radioactive distribution in the whole body. For example, substantial inter-tissue variances in the further radiometabolism of FDG, i.e., away from FDG-6-phosphate, have been demonstrated ex vivo in different rat organs [21]. Moreover, a relative tissue-dependent distribution of individual radiometabolites replicating the physiological features of the respective organs has also been established [21]. Therefore, by understanding the occurrence and biochemical nature of different radiometabolic compounds, it is possible to gain extensive insight into the pathological and physiological processes by PET. To calculate physiological parameters such as receptor density and metabolic rates, it is necessary to know the concentration of the tracer and its radiometabolites in the blood and tissues over time. Hence, it is vital to establish methods by which radiometabolites, especially those having the potential to cross the BBB, can be identified and measured over time. Identification of radiometabolites during the evolution of a new radiotracer is also important as it offers information about the most suitable position to radiolabel a compound to escape any production of inquisitive radiometabolites [22, 23]. There are some reviews already published on PET radiometabolites [24, 25]. However, those reviews mainly focus on the sample preparation techniques and recently available technology to improve the radiometabolite analysis process. Our review mainly summarizes the chemical and structural identities of radiometabolites of different radiotracers. Moreover, this review also highlights how a slight chemical modification could reduce the formation of radiometabolites, which could interfere with the results of PET imaging.

## Importance of PET radiometabolite analysis

Radiometabolite adjusted arterial input function is crucial for the exact quantification of the PET radiotracers that do not possess any appropriate reference region in the brain. There are two main reasons for the chemical identification of radiometabolites: firstly, to determine the amount of parent radiotracers in plasma, and secondly, to rule out or correct any radiometabolite accumulation in peripheral tissue. Radiometabolite formations of the tracer might be a concern for the PET study, as radiometabolic products may display considerably contrasting administration patterns into the body, as compared to the tracer itself. An understanding of radiometabolite formation and its chemical nature may provide important information on physiological, biochemical, and pathological processes by PET. To fulfill this requirement, data analysis models were established to evaluate the correlation between the acquired data and the biophysical factors influencing radiotracer accumulation and biotransformation [26, 27]. Moreover, radiotracer metabolism can significantly influence the arterial input function, which is a factor applied for analyzing PET images quantitatively. Radiometabolite examinations in the plasma are a crucial requirement for the precise assessment and revelation of PET imaging and the determination of pathological changes in metabolic pathways [28]. After estimating the radioisotopic decay and restructuring it into an image, the exact biodistribution of the compound is accessible. Hence, radiometabolite analysis is an extremely important topic in PET imaging. In some cases, it was noticed that a radiometabolite can show a better affinity for the target than its parent radiotracer. For example, [^11^C]Loperamide and its putative radiometabolite [*N*-methyl-^11^C]*N*-desmethyl-loperamide both behave as the avid substrate for P-gp [29, 30]. Actually, [*N*-methyl-^11^C]*N*-desmethyl-loperamide could be superior to [^11^C]Loperamide in determining P-gp function at the blood–brain barrier because further demethylation of [*N*-methyl-^11^C]*N*-desmethyl-loperamide will generate a more polar radiometabolites that have negligible access into the brain [31]. There are some instances where the parent radiotracer and its radiometabolites both have the affinity for the same targets. In this case, the cold compound of the radiotracer can be injected for checking their competition for the target. For example, both [^18^F]LBT-999 and its radiometabolite (2β-carbomethoxy-3b-(4′-tolyl) nortropane) have the binding affinity for dopamine transporter (DAT) [32]. In a PET experiment, Saba et al. have shown that after intravenous injection of cold metabolite, it induced a strong displacement of [^18^F]LBT-999 in the striata, suggesting that the radiometabolite can cross the BBB and competing with the radiotracer [^18^F]LBT-999 for the target [33].

## Radiotracer metabolism

Most of the drug/tracer metabolism takes place in the liver. However, it may also take place in the kidney or intestine. Usually, metabolism occurs to transform the radiotracer into a compound that is more water-soluble and easily excreted. Similar to the drug, the radiotracer metabolism also takes place in two phases. In phase I, the radiotracers are chemically changed by enzymes through oxidation, reduction, and hydrolysis. Hence, in phase I, a new functional group is generated which can be used in the next phase. In phase II, some hydrophilic moiety is attached to that functional group to form readily water-soluble conjugates that can be easily excreted through urine [34, 35]. Table 1 summarizes the different processes, substrates, and products of phase I and phase II.

**Table 1:** Phase I and phase II metabolism of the radiotracer

Phase I metabolism			Phase II metabolism		
Process	Substrate	Product	Process	Substrate	Product
Oxidation	Aromatic compounds Aliphatic compounds Aromatic ethers Aromatic amine Tertiary Amine	Hydroxylated compound Alcohol, aldehyde, ketones, acids Phenols and Aliphatic aldehydes Hydroxyamino compound *N*-Oxide	Glucuronidation	Alcohol or phenol derivatives of the radiotracers and glucuronic acid	Conjugated Ester
Reduction	Aldehydes, ketones Carbon–carbon double bond Nitro group	Alcohols Corresponding saturated compounds Corresponding amine	Sulphonation	Alcohol and phenol derivatives of the radiotracers and its metabolites	Conjugated product
Hydrolysis	Ester, amide	Acid	Acetylation	Aliphatic or aromatic amine	Amides

## Methods of radiometabolite analysis

Several chromatographic techniques including thin-layer chromatography (TLC) [36], high-performance liquid chromatography (HPLC) [37], micellar chromatography [38], and overpressure thin-layer chromatography (OPTLC) [39] were commonly used for the examination of radiometabolites. Among these techniques, TLC was the earliest known chromatographic tool applied in radiochemistry to separate radioactive compounds. To date, TLC is an important technique and is regularly used in radiopharmacy departments, to determine the radiochemical purity of radiopharmaceuticals. This technique is very easy to apply, fast, inexpensive, and most importantly it can detect all deposited radioactivity without losing any information [40]. However, TLC, generally a qualitative technique, suffers from several limitations including resolution, accuracy, and reproducibility [41]. Therefore, TLC is not a suitable tool for radiometabolite analysis. In comparison to TLC, the OPTLC comes with superior resolution and rapid analysis. Until recently, the most widely used technique for the analysis of radiometabolites of a radiotracer is the HPLC, combined with radioactivity detectors. HPLC analysis is reproducible, and it allows to quantify narrow, rider, and shoulder peaks precisely which is not possible for TLC. A multistep sample extraction process is required before it could be injected into the HPLC system [42]. However, due to the reasonably lengthy preparation procedure in combination with the very short half-lives of the radiotracers, loss of information may occur particularly at the later stages of the PET imaging experiments. Additionally, multi-stage blood extraction may lead to a decrease or alteration of information. This may induce bias and hamper the repeatability of the analysis. To get rid of this time-consuming sample preparation technique, solid phase extraction (SPE) of the radiometabolite can serve as a suitable choice. In SPE, first, the plasma sample is loaded into an SPE cartridge directly or after the precipitation of protein. Then the radiotracer and its metabolites are extracted from SPE depending on their polarity. Although direct blood sampling could largely reduce the sample preparation time, it is still a challenging issue in radiometabolite analysis. Another important and well-known approach for radiometabolite analysis is column-switching HPLC. Here, the plasma sample is directly loaded into a capture column which is a kind of short SPE cartridge. Following this, the radiotracer and its metabolites are eluted into a reverse-phase column of a radio-HPLC [43–45]. This semi-automated method effectively reduces time-consuming sample preparation steps and exposure to the radioactivity with better reproducibility. However, this method suffers from high back pressure as it is required a large plasma volume for radiometabolite analysis. Nakao et al. have developed an alternative rapid and efficient method based on micellar liquid chromatography for the direct analysis of plasma radiometabolites [24, 38, 46]. This method uses micellar/high submicellar liquid chromatography along with semi-preparative alkyl-bonded silica column for the analysis of radiometabolites. The steep gradient and high flow rate are used for the rapid removal of the macromolecule from plasma. Compared to the particle-based column, the monolithic column provides high resolution and better sensitivity. The main advantage of this approach is that it reduces time-consuming sample preparation steps. Besides, it also allows the analysis of a large number of samples with appropriate sensitivity. Currently, protein precipitation and SPE of radiometabolites are the two most encouraging sample preparation procedures designed for radioactive metabolite analysis. Table 2 summarizes the various advantages and limitations of different analytical techniques applied to radiometabolite analysis.

**Table 2:** Advantages and limitations of different analytical techniques applied to radiometabolite analysis

Analytical techniques	Sensitivity	Resolution	Measurement speed	Required pre-treatment	Cost
Radio-TLC	High	Low	Slow	Yes	Low
OPTLC	High	Moderate	Fast	Yes	Moderate
Radio-HPLC	High	High	Fast	Yes	High
Column-switching HPLC	High	High	Fast	No	High
SPE	Moderate	Low	Fast	Usually no	Low
Micellar chromatography	High	Low	Fast	No	Low

## Sample preparation

The most commonly used biological samples for the radiometabolite analysis are blood, plasma, and tissue. The direct analysis of these samples has several limitations. Hence, most of the time, it is essential to separate the radiotracer and its metabolites from the biological matrix.

### Disruption of tissue samples [19]

It is well known that the ease of sample analysis increases with the degree of fluidity. Hence, all the tissue samples are usually required to go through some mechanical procedures before the sample is analyzed [19]. There are some common techniques used for the disruption of tissue samples including “motor and pastel” technique, blades technique, and sonication techniques. One of the disadvantages of these procedures is that during this procedure the radiotracers or its metabolites may change the chemical structure. Sometimes these samples produce emulsion, and because of it, subsequent sample analysis may be difficult.

### Protein elimination

**Fig. 1:**
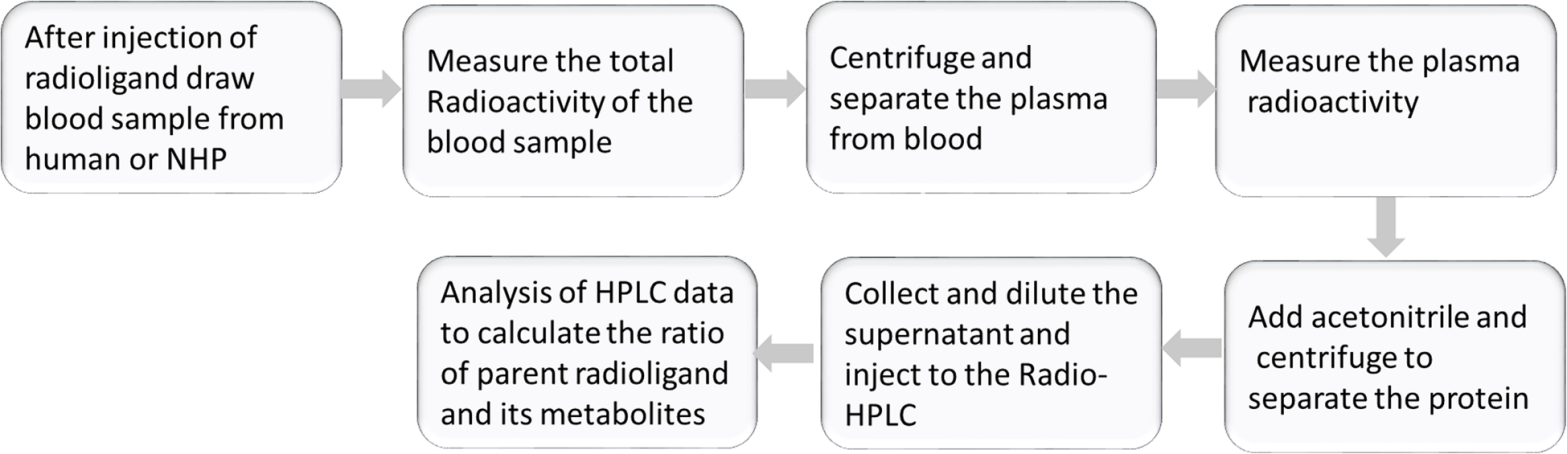
The schematic diagram of protein elimination from the blood sample

The biological samples or disrupted tissue samples usually have a large amount of protein. Sometimes these proteins bind very strongly to the radiotracers or its radiometabolites. To examine the radiometabolites, it is required to separate the radiotracers and its radiometabolites from the protein [25]. Besides, multistep manual extraction of blood samples is necessary to remove the biological matrix before injection of the sample into the HPLC system, and in this way, the risk of HPLC column blockage can also be minimized. Hence, the blood sample is required to be centrifuged. The plasma attained from this is then mixed with an organic solvent such as acetonitrile to precipitate plasma proteins. This mixture is then centrifuged again to collect the organic fraction as the supernatant [42]. The collected supernatant, which contains the desired compound is then injected into the HPLC instrument. The protein precipitation techniques from the blood sample can be described by the schematic diagram shown in Fig. 1.

## Importance of plasma protein binding analysis

One of the most important aspects of PET study is to find out the amount of free fraction of an injected radiotracer in plasma. After the injection of the radiotracer into the body, a fraction of it binds to the plasma protein. The remaining fraction (free radiotracer) is then making an equilibrium with its plasma protein bounded fraction [42]. It is worth mentioning that only the free fraction of the radiotracer is available to bind the target of interest and the efficacy of a drug depends on the free fraction present in plasma [47]. When the two drugs were administrated into the body at the same time, it is particularly important to know their plasma protein binding affinity as one drug can replace a proportion of other drugs from being bound to the plasma protein, enabling a greater proportion of free drug fraction. The methods to measure the amount of plasma protein binding and the free fraction of radiotracer were discussed in detail by Moein et al [25].

## Case studies

### Radiometabolite analysis of [^11^C]PBB3

In 2015, Kazunori Kawamura et al. revealed the structure of the key radiometabolites of [^11^C]PBB3 by high-performance liquid chromatography-mass spectrometry (HPLC-MS) and suggested that its possible metabolic route is through in vitro, in vivo, and in silico investigations [48]. PBBs, a family of pyridyl and phenyl-butadienyl-benzothiazoles compounds, were initially examined by Makoto Higuchi et al. for the applications of prospective imaging radiotracers of tau deposition in the brain [49, 50]. [^11^C]PBB3 appeared as the most promising candidate among all the PBB compounds. It has a large affinity for tau protein aggregates with a *K*_d_ value of 2.55 nM and the selectivity of PBB3 for tau is around 50 times higher in comparison to Aβ deposits [49]. In another study, the group also revealed that [^11^C] PBB3 readily degraded to form a polar radiometabolite [49, 51] and thus potentially impeding a simplified PET image analysis. An identical radiometabolite was also obtained from the mouse brain after the administration of [^11^C]PBB3 as the PET radiotracer.

**Fig. 2:**
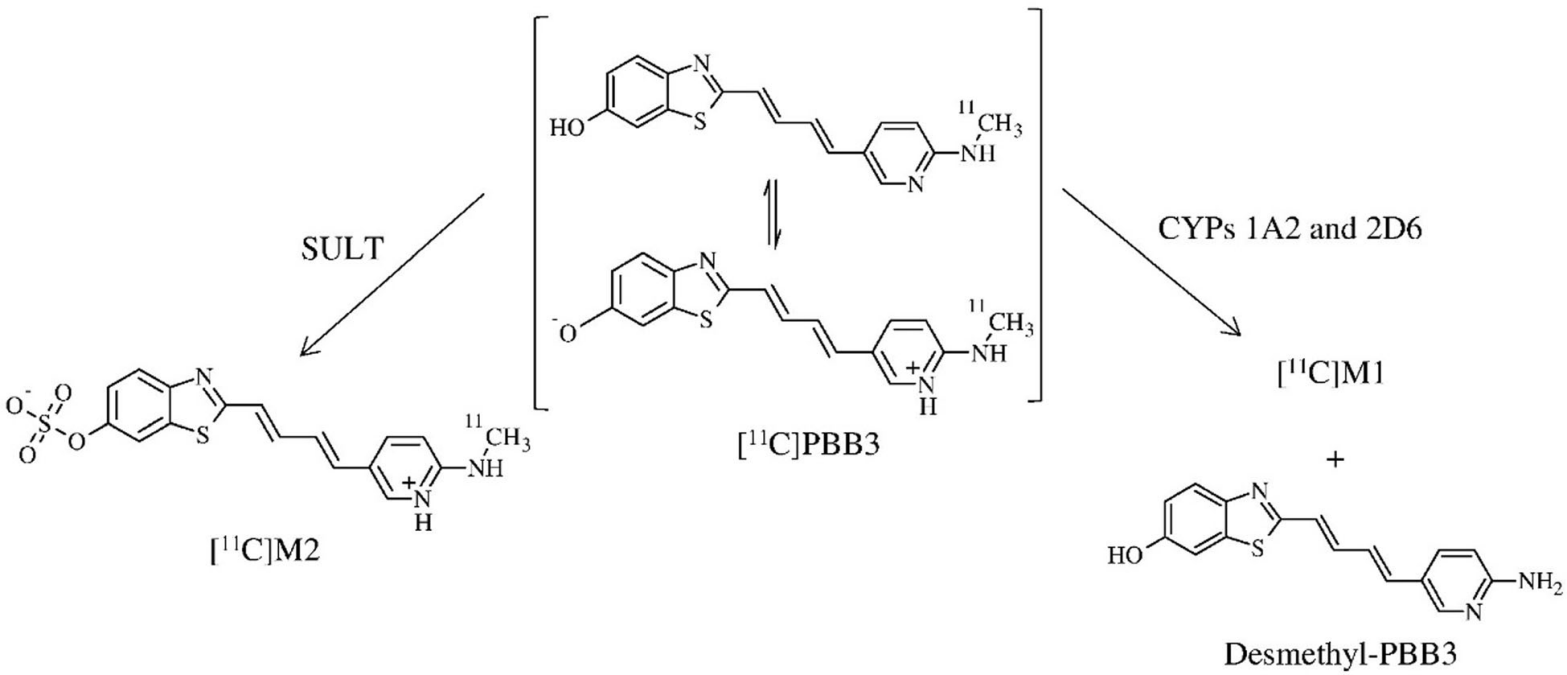
Major metabolic pathways for [^11^C]PBB3 and chemical structures of metabolites (SULT sulfotransferase). Adapted with permission from Ref. [48]

**Fig. 3:**
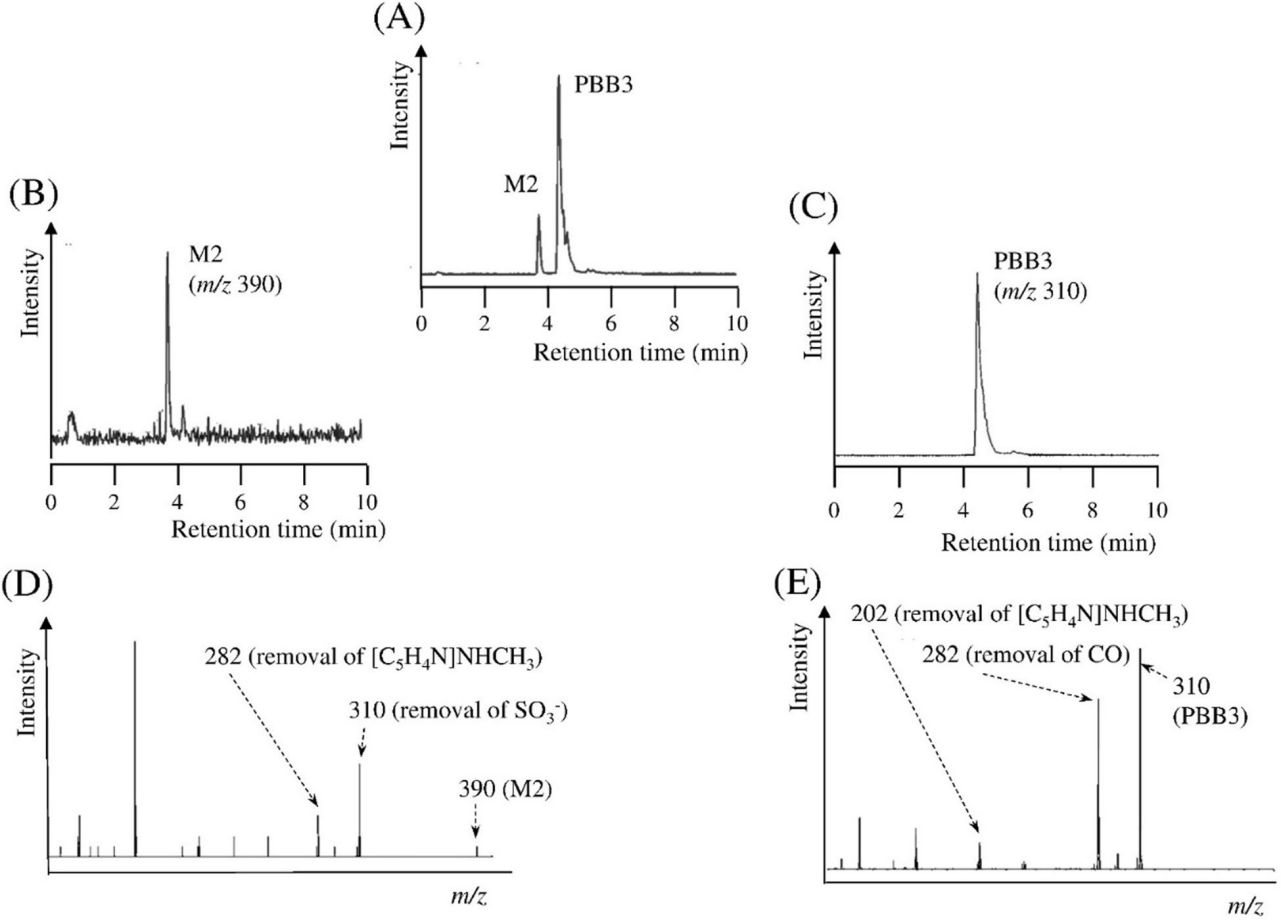
**(a)** Mass chromatograms of in vivo metabolite analysis using carrier-added [^11^C]PBB3 in mouse plasma. Enhanced product ion chromatograms of the major metabolite M2 (*m/z* 390, (**b**) of PBB3 and PBB3 (*m/z* 310, (**c**). MS–MS fragmentation patterns of M2 (**d**) and PBB3 (**e**). Adapted with permission from Ref. [48]

In vivo radiometabolite examination demonstrates that the fraction of parent [^11^C]PBB3 is around 30%, 5 min after the administration of [^11^C]PBB3 (carrier-added) in mouse plasma was relatively larger than the results obtained (1.9%) after administration of [^11^C]PBB3 alone [51]. The results ascertained that extra amounts of carrier PBB3 reduce the metabolic rate of [^11^C]PBB3. Analytical radio-HPLC offers two peaks corresponding to the two radiometabolites and parent [^11^C]PBB3 peaks in the in vivo metabolite examination of [^11^C]PBB3 (carrier-added) mouse plasma. A small radioactive metabolite peak ([^11^C]M1) at 0.6 min was assumed to have formed due to the demethylation of the radiotracer in the presence of cytochrome P450 enzymes (CYPs) (Fig. 2) [48]. The mass of the main metabolite ([^11^C]M2) which appears at 1.6 min was obtained as 390 [M+H+] from LC–MS (Fig. 3b) [48]. Hence, the main metabolite with *m*/*z* 390 was non-identical from the one formed in the phase I radiometabolism facilitated by CYPs [52, 53]. The in vitro radiometabolite examination of [^11^C]PBB3 by incubating with a mouse as well as a human liver S9 fraction followed by the evaluation of this result with the in vivo radiometabolite analysis of mouse plasma shows that the main radioactivity peak was equivalent to [^11^C]M2 with *m*/*z* 390, produced due to the sulfate conjugation of [^11^C]PBB3 facilitated by a sulfotransferase enzyme. The incubation of [^11^C]PBB3 with mouse liver microsomes (MLM) and human liver microsomes (HLM) having a CYP activator confirms that the minor radiometabolite is [^11^C]M1, which was produced by the CYP facilitated demethylation.

Only a small variation between mouse and human samples were detected for the radiometabolism of [^11^C]PBB3 (in vitro) by cytochrome P450 and sulfotransferase enzymes. Therefore, [^11^C]PBB3 metabolism in mice may offer a substantial understanding of the pharmacokinetic behavior of the radiotracer with PET imaging in humans. These results have established that the radiotracer [^11^C]PBB3 mostly metabolized to produce [^11^C]M2 a sulfate conjugation reaction facilitated by sulfotransferases enzymes and a negligible radiometabolite, [^11^C]M1, was produced due to oxidation being interposed by the CYPs (Fig. 2). Hence, it is extremely important to determine the metabolic pathway and radiometabolite structure for assessing the clinical usage of [^11^C]PBB3. Besides, the information also could be used in designing new PBB analogs which are more efficient and biostable than the parent compound.

### Radiometabolite analysis of [^11^C]flumazenil

**Fig. 4:**
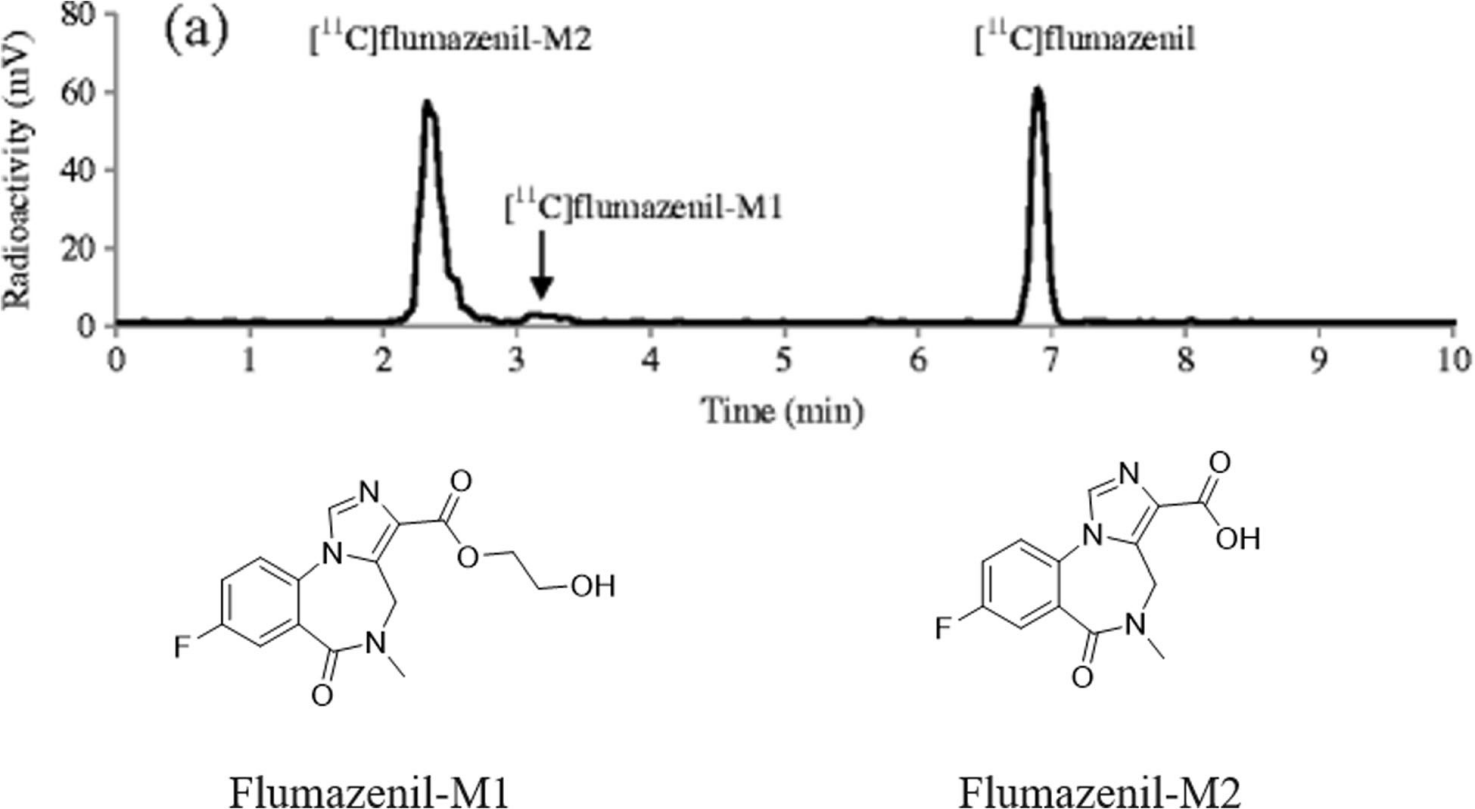
Fast radio-LC chromatograms of (**a**) rhesus monkey plasma collected 20 min after administration of [^11^C]flumazenil. Adapted with permission from Ref. [54]

In 2013, Amini et al. presented a strategy to find out radioactive metabolites in monkey plasma after the injection of [^11^C] flumazenil, a PET radiotracer—for GABAA receptor [54]. Utilizing this procedure, radiometabolites formed in vivo could be acknowledged and studied by the support of fast radio-LC, ultra high-performance liquid chromatography/quadrupole-time of flight-mass spectrometry (UHPLC/Q-ToF-MS), and hepatic microsomes. According to this strategy, the radiometabolites were first formed in vitro by liver microsomes as radiotracers are mostly metabolized in the liver. All the radiometabolite peaks were successively separated and collected independently by fast radio-LC and identified by UHPLC/Q-ToF-mass spectrometry. The in vivo metabolic profiles are then related to and recognized by the help of the in vitro results. When the in vitro radiometabolism of [^11^C]flumazenil was analyzed by fast radio-LC, it offered three peaks corresponding to [^11^C]flumazenil and its two radiometabolites, -[^11^C]flumazenil-M1 and [^11^C]flumazenil-M2. Mass spectrometry analysis offers monoisotopic masses of [^11^C]flumazenil, its radiometabolites, and their fragmented ions. In mass spectrometry, the flumazenil fragmentation pattern gives an acylium ion peak corresponding to *m*/*z* 258.0691. This result is compared to the one reported previously [55]. The two radiometabolites of flumazenil were identified—one is a hydroxyl-ethyl ester (flumazenil-M1) and the other one is an acid (flumazenil-M2). These results demonstrate that the identical fragmentation of flumazenil occurs due to radiometabolism. To identify the in vivo metabolism of [^11^C]flumazenil, after administration of the radiotracer, samples from the rhesus monkey plasma were collected at intervals and examined by fast radio-LC (Fig. 4) [54]. When the metabolic stability of [^11^C] flumazenil in vitro (no carrier-added) as well as in vivo was examined, the radiotracer metabolism rate was found to be faster in in vivo than in in vitro. Nevertheless, both in vitro and in vivo experiments produce an equal number of radiometabolites and in both cases, [^11^C]flumazenil-M2 appears to be the major radiometabolite. A similar metabolic profile was reported in primates and human plasma [56–59]. There were no metabolites detected in the absence of any microsomes in in vitro. These results demonstrate the microsome dependency of radiometabolite formation of [^11^C]flumazenil. The most important metabolite of [^11^C]flumazenil is an acid, and it does not penetrate the blood–brain barrier in normal human subjects [60]. Besides, it has negligible in vitro human benzodiazepine receptors binding affinity. Hence, the radioactive metabolite could only marginally impact the estimation of free ligand concentration in the brain and the interpretation of the PET data.

### Radiometabolite analysis of [^11^C]PBR28

**Fig. 5:**
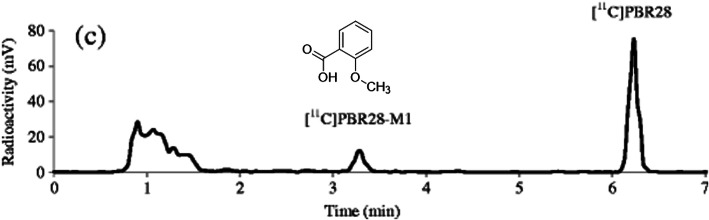
Fast radio-LC chromatograms of human plasma collected 10 min after administration of [^11^C] PBR28. The radioligands and their identified radiometabolites are marked on each chromatogram. Adapted with permission from Ref. [54]

[^11^C]PBR28, a PET radiotracer for an18-kDa translocator protein is widely used in brain imaging [61]. Carrier-added solutions of [^11^C]PBR28 were incubated with human liver microsomes to find out its phase I radiometabolism [54]. The metabolism reaction was paused at different intervals and the progress of radiometabolite formation was examined using fast radio-LC. The radiotracer peak and the produced radiometabolite peaks were isolated and recognized by mass spectrometry. The mass spectrometry data suggested that the PBR28-M1 was produced by two-step biotransformation of PBR28. Initially, N-debenzylation of PBR28 following the oxidation of [^11^C]2-methoxybenzaldehyde produces [11C]2-methoxybenzoicacid (PBR28-M1). The identity of the radiometabolite was additionally established by matching its retention time with the reference standard of PBR28-M1. PBR28-M1 was also documented earlier in vivo rats’ studies [61]. The in vivo metabolite identification was demonstrated by comparing the retention times of its in vitro metabolite peaks. In the case of the in vivo radiometabolism study, after the injection of [^11^C]PBR28 in a human, the plasma samples were acquired at multiple time intervals and examined with standard radio-LC and fast radio-LC (Fig. 5) [54]. The examination of the metabolic stability of the [^11^C]PBR28 radiotracers confirmed that they are metabolized faster in in vitro than in in vivo. Interestingly enough, four additional radiometabolite peaks were observed when [^11^C]PBR28 was incubated with the human liver microsome. However, these peaks are missing in human plasma. Although the above strategy can be successfully applied to identify the chemical form of several radiometabolites, it is quite difficult to foresee the comparative quantity of radiometabolites formed in in vivo with the help of the in vitro route.

### Radiometabolite analysis of [^18^F]FEPE2I

**Fig. 6:**
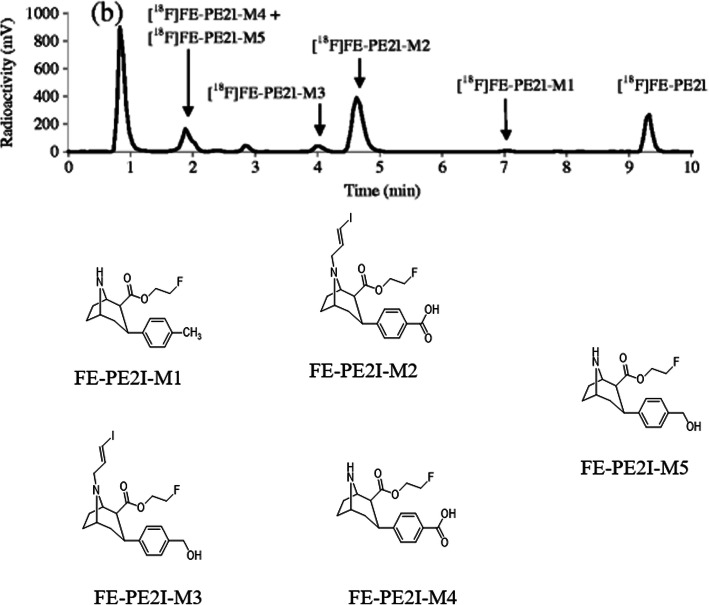
Fast radio-LC chromatograms of rhesus monkey plasma collected 20 min after administration of [^18^F]FE-PE2I. Adapted with permission from Ref. [54]

[^18^F]FEPE2I which targets the DAT is a PET radiotracer used in neuroimaging [62]. Phase I radiometabolites of the PET radiotracer [^18^F]FE-PE2I was investigated by the incubation of carrier-added solutions of the radiotracer with microsomes from a monkey liver. Five major radiometabolites were identified due to the different kinds of in vitro enzymatic biotransformations, including N-dealkylation and benzylic hydroxylation followed by additional oxidation of benzyl alcohol, the primary metabolite. The study also demonstrates the in vivo radiometabolism by analyzing the plasma samples from a rhesus monkey at different time points after the administration of [^18^F]FE-PE2I by using conventional radio-LC and fast radio-LC. Seven different radiometabolite peaks were separated with the help of a fast radio-LC and among them, five were recognized. However, only two radiometabolite peaks were witnessed by the conventional radio-HPLC as reported elsewhere [63, 64]. When examining the metabolic stability of [^18^F]FE-PE2I, it was observed that the rate of radiometabolism is faster in the case of in vitro than in vivo. Although equal numbers of radioactive metabolites were identified both in in vitro as well as in in vivo analysis, the major radiometabolite amount significantly varies between the two studies. In the case of in vivo, the major radiometabolite was demonstrated as [^18^F]FE-PE2I-M2 (Fig. 6) [54], whereas the combined peak of [^18^F]FE-PE2I-M4 and [^18^F]FE-PE2I-M5 is appeared to be the predominant fraction in in vitro analysis. Similar kinds of variations between in vivo and in vitro radiometabolites formed are also observed in other studies [65, 66].

### Radiometabolite of [^11^C]MADAM

**Fig. 7:**
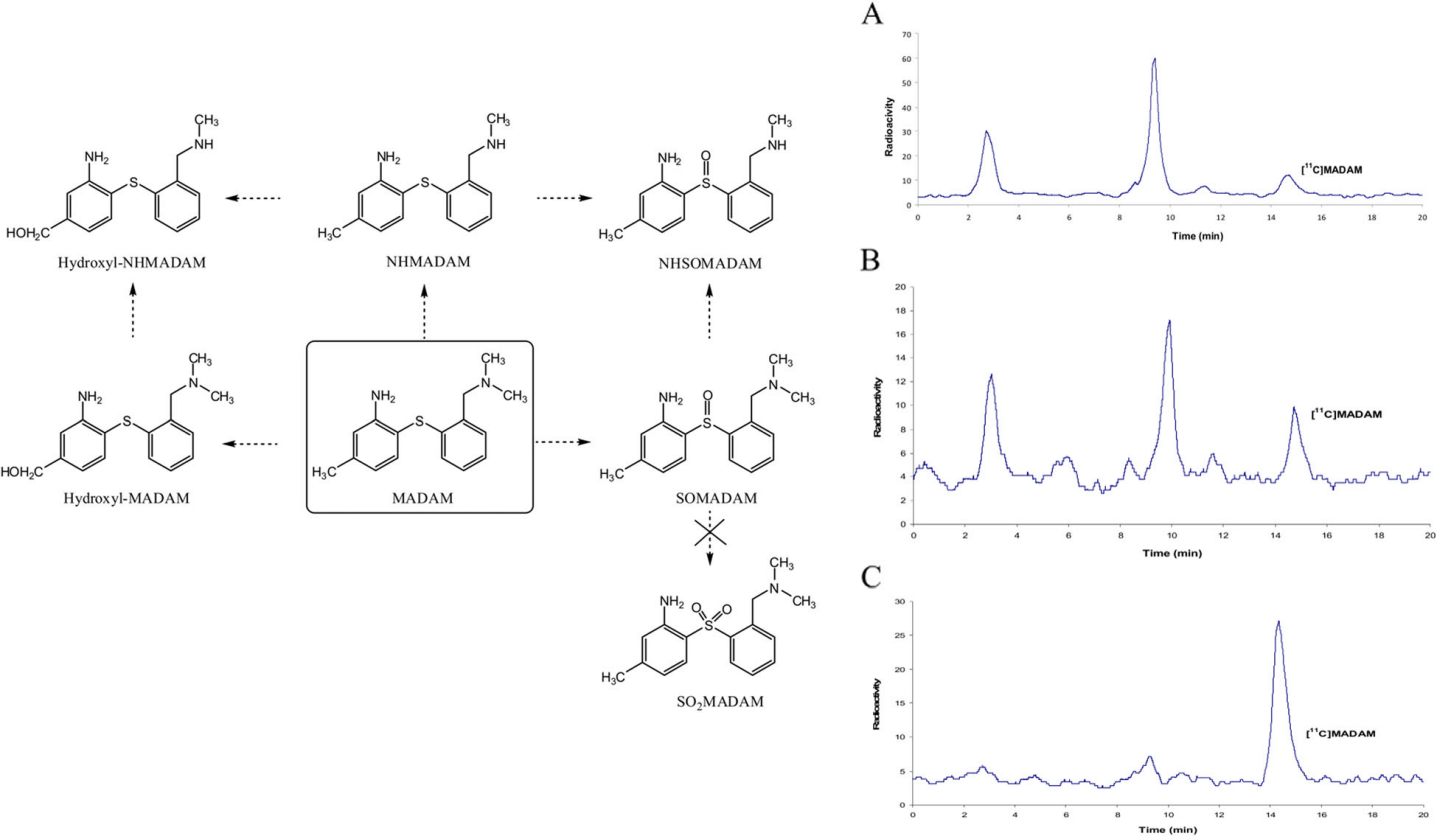
Proposed in vitro metabolic pathways of MADAM. Radio-HPLC chromatograms of rat urine samples after perfusion of (**a)** [^11^C]MADAM, (**b)** [^11^C] MADAM /MADAM (25 μg), and (**c)** [^11^C]MADAM /MADAM (125 μg) for 15 min. Adapted with permission from Ref. [69]

Diphenyl sulfide derivatives have proven to be potential PET/SPECT radiotracers for serotonin transporter (SERT) study in vivo and are thus extremely crucial to examine their radioactive metabolism [67, 68]. Christer et al. had explored the metabolic portfolio of [^11^C]MADAM, a widely used diphenyl sulfide-based radiotracer for PET-SERT examination in vivo [69]. [^11^C]MADAM/MADAM metabolism was demonstrated with liver microcosm from a rat (RLM) and a human (HLM) in association with radio-HPLC or UHPLC/Q-ToF-MS. The study examined the influence of the carrier on the rate of metabolism of [^11^C]MADAM both in in vitro as well as in vivo with the help of radio-HPLC. To identify the carrier concentration dependency of metabolite formation in vitro, two different concentrations of RLM and HLM were incubated with [^11^C]MADAM. The urine samples were also examined with radio-HPLC (Fig. 7) [69]. The results suggest that the metabolites were produced in in vitro because of the following biotransformation: N-benzylic hydroxylation, S-oxidation, and demethylation (Fig. 7) [69]. Their study also revealed that the carrier presence significantly enhanced the [^11^C]MADAM metabolism rate in RLM/HLM in in vitro experiments as well as in in vivo rat studies. The decent commonality of the outcomes anticipated by RLM/HLM experiments with the data acquired from the rat studies in vivo designates that [^11^C]MADAM metabolism rate is dose dependent. Therefore, this matter requires to be examined when the diarylsulfide compounds are applied for the quantifications of SERT by PET.

### Radiometabolite analysis of (+)-[^18^F]flubatine

**Fig. 8:**
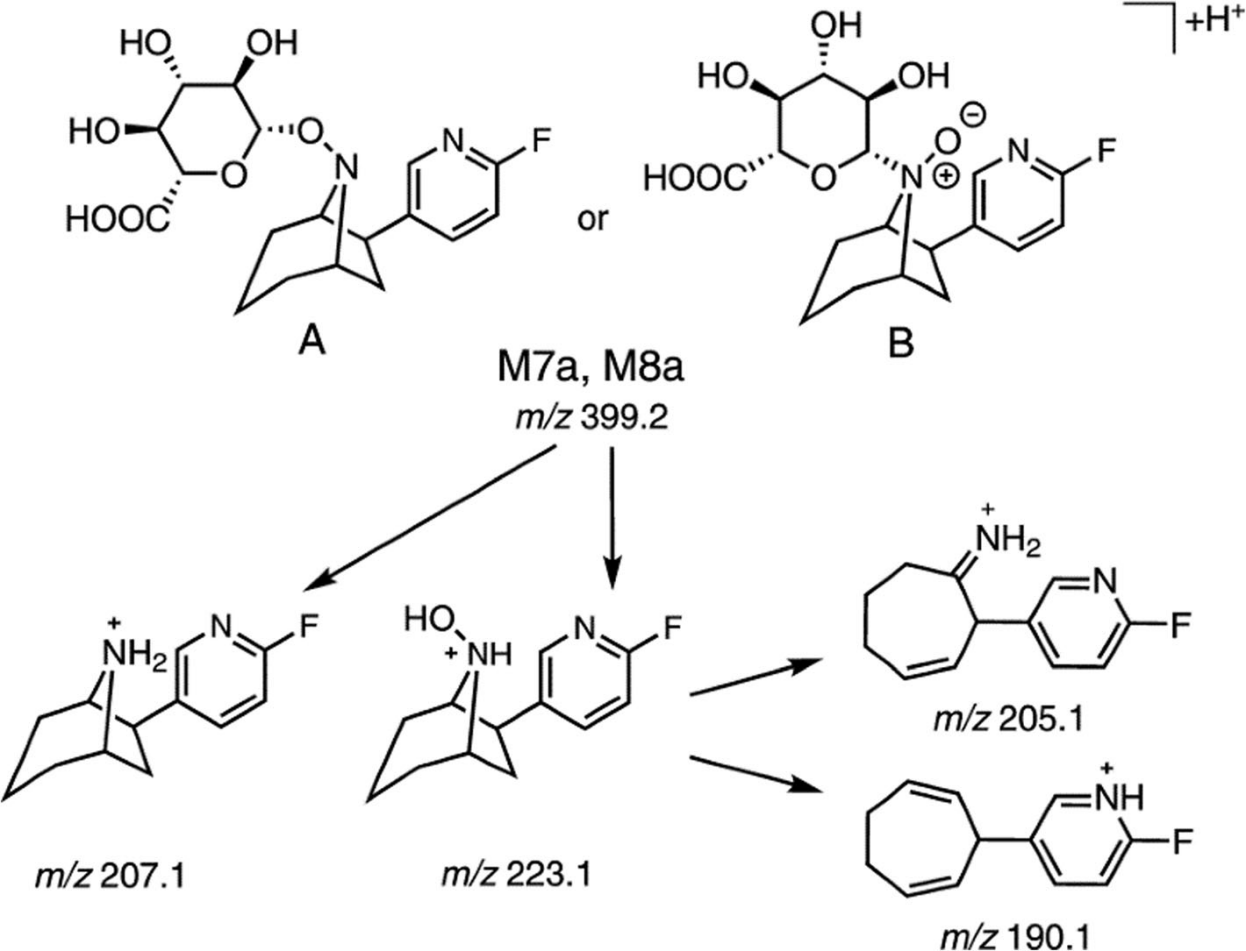
Proposed structures and fragmentation pathways shown for glucuronide conjugates M7a and M8a, drawn as N-*O*-glucuronide (A) and N+(O−)-glucuronide (B); similarly, for enantiomers M7b and M8b. Adapted with permission from Ref. [71]

**Fig. 9:**
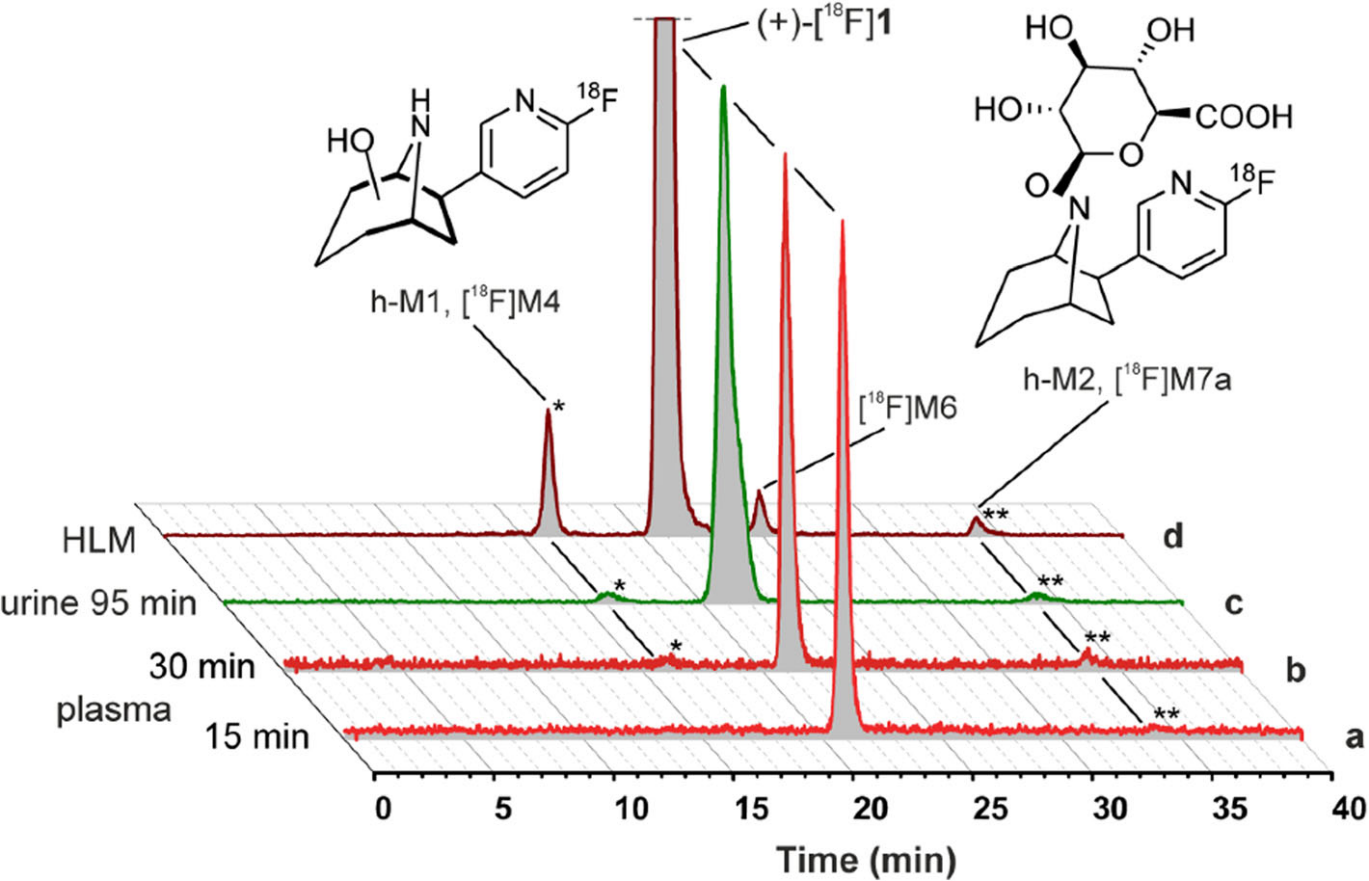
Comparison of metabolic profiles of (+)-[^18^F]1 (in vivo vs. in vitro) and identification of radiometabolites. Representative radio-HPLC chromatograms of samples obtained from human subjects: (a) plasma, 15 min; (b) plasma, 30 min; (c) urine, 95 min after injection; and (d) after HLM incubation (NADPH, UDPGA, TRIS, pH 8.4, 37 C, 120 min). Scaling was adjusted for each chromatogram. Adapted with permission from Ref. [71]

In the PET study, both the enantiomers of [^18^F]flubatine are applied as a neuroimaging agent for α_4_β_2_nicotinic acetylcholine receptors (nAChRs) [70]. In 2018, Peter Brust et al. demonstrated a general strategy for identifying radiometabolites of (+)-[^18^F] flubatine formed in humans by studying the radiometabolism in pigs (in vivo) and incubations with liver microsomes (in vitro) assisted with radio-HPLC and LC-MS/MS [71]. Chromatographic investigations of both urine and plasma samples from humans displayed the presence of very high fractions of (+)-[^18^F]flubatine. Almost 96% of (+)-[^18^F]flubatine stayed unaffected after 30 min of injection. The enantiomer of the (+)-[^18^F]flubatine (i.e., (−)-[^18^F]flubatine) also showed a large fraction of the unaffected radiotracer, which was around 91% [72] and 93% [73] at the time of the investigation. The (+)-[^18^F]flubatine undertook a biochemical transformation via *C*-hydroxylation within the azabicyclic ring to produce h-M1, which is equivalent to [^18^F]M4 produced in vitro (Fig. 8) [71]. The bridgehead nitrogen (*N*8) hydroxylation (*N*8) followed by a conjugation reaction by glucuronic acid leads to the formation of a phase II radiometabolite h-M2, which is the same as [^18^F]M7a, produced in vitro. Hence, the radioactive metabolites spotted in humans was in decent accordance with those produced by the human liver microsomes (Fig. 9) [71]. The structures of h-M1 as well as also h-M2 are highly lipophobic and hence have a lesser chance to pass through the blood–brain barrier. Thus, the result was in agreement with the metabolic examination done with the mice where an extremely small amount of radioactive metabolites (< 7%) were spotted in the brain, after 30 min of administration [74]. The radiometabolism of the (−)-[^18^F]flubatine was also examined in the in vivo preclinical studies [75–77] as well as in humans [72, 73, 78]. However, no structural information about the radiometabolites of (−)-[^18^F]flubatine has been reported yet. In a clinical study, a radiometabolite was noticed in very small amounts by the radio-HPLC, but no characterization study was performed [76]. This metabolic product is mostly formed due to the *N*-hydroxylation of the (−)-[^18^F]flubatine, followed by a glucuronidation as identified for the (+)-[^18^F]flubatine. As the amount of radiometabolite formed is too little, the radiometabolism of the (+)-[^18^F]flubatine does not need to be considered in the narrative of the arterial input function for the analysis of PET data through kinetic modeling. Because of the little formation of these radiometabolites in humans, the (+)-[^18^F]flubatine displays a great metabolic stability in the clinical study. Thus, the (+)-[^18^F]flubatine has been accepted as a highly suitable radiotracer for the PET imaging of α_4_β_2_ nAChRs.

## Improvement of metabolic stability

Improvement of the metabolic stability of the radiotracer is as important as the analysis and identification of its radiometabolites. An increase in metabolic stability could improve the quality of PET imaging results. There are two major ways to improve the metabolic stability of the radiotracer. The first approach is to substitute the hydrogen present in the reactive site by deuterium. The deuterium substitution of the methyl or ethyl group will usually reduce the rate of metabolism as it requires higher energy to cleave a carbon–deuterium covalent bond other than the carbon–hydrogen bond simply due to a higher mass effect of deuterium. The second approach is to change the labeling position without altering any of the structural or chemical changes of the parent radiotracer. In this section, we will look into the case study for the above two approaches using [^18^F]Fluorocholine and [^11^C]WAY-100635, respectively.

### Stability of [^18^F]fluorocholine vs [^18^F]fluoro-[1,2-2H4]choline

**Fig. 10:**
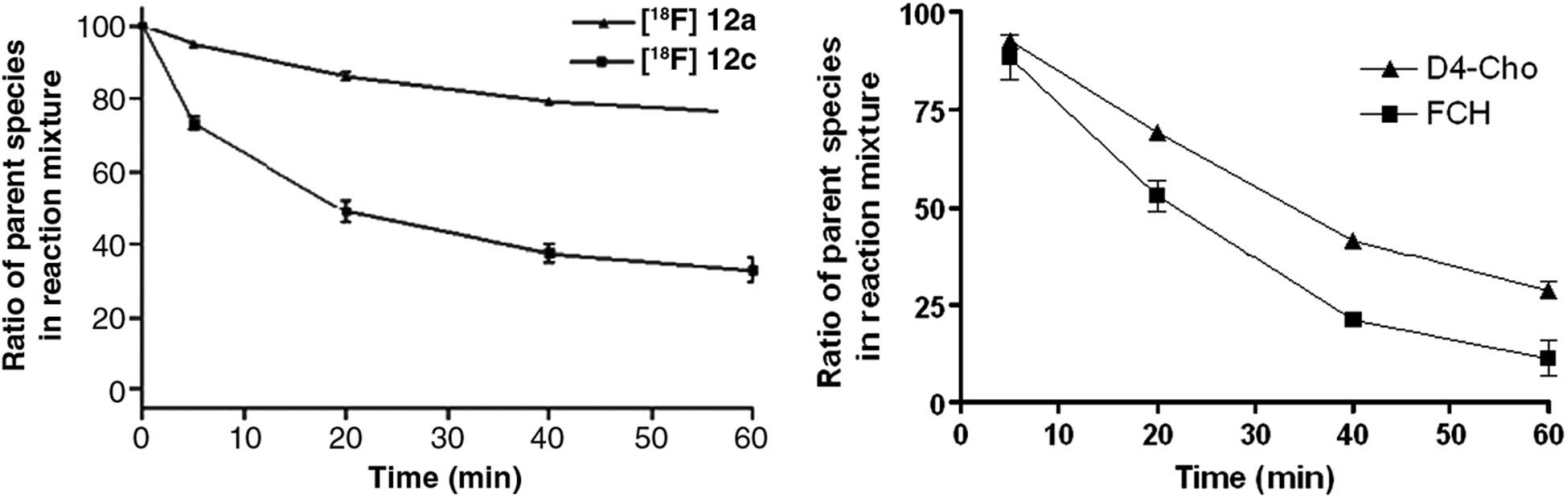
**a** Chemical oxidation potential of [^18^F]fluorocholine (12a) and [^18^F] fluoro-[1,2-2H4]choline (12c) in the presence of potassium permanganate. b Time-course stability assay of [^18^F]12a and [^18^F]12c in the presence of choline oxidase demonstrating the conversion of its parent compounds to their respective betaine analogs. Adapted with permission from Ref. [81]

Choline radiotracers are commonly applied for clinical PET imaging in oncology [79, 80]. In 2011, Smith et al. reported a unique di-deuterated choline radiotracer, [^18^F]fluoro-[1,2-2H4]choline, and assessed its in vitro and in vivo stability as well as its potential use as a PET imaging radiotracer [81]. Chemical oxidation of these radiotracers by the chemical potassium permanganate shows that [^18^F]fluoro-[1,2-2H4]choline has relatively higher stability to oxidation when compared to [^18^F]fluorocholine, which was expected from the isotope effect (Fig. 10a) [81]. Radio-HPLC profiles indicate the presence of almost similar amounts of the original radiotracers and the major radiometabolite, betaine for [^18^F]fluorocholine after incubation with potassium permanganate for 20 min. However, only a trace amount of betaine was observed for [^18^F]fluoro-[1,2-2H4]choline in the same conditions. Oxidative stability of these radiotracers was also examined against the enzyme choline oxidase (Fig. 10b) [81], and this indicates a greater degree of oxidation compared to the potassium permanganate system. However, both enzymatic oxidation and in vitro chemical examination of [^18^F]fluorocholine and its di-deuterated analog directed that the later have higher oxidative resistance than [^18^F]fluorocholine. This in vitro result is also supported by the in vivo radiometabolite profile. The radio-HPLC exploration of plasma samples provided some additional indication for an increase in the resistance of the [^18^F]fluoro-[1,2-2H4]choline against oxidation relative to the [^18^F]fluorocholine. In the case of [^18^F]fluorocholine, simply a little portion of the original radiotracer was detected in the radio-HPLC profile. However, a considerable peak in the original radiotracer was still spotted in the plasma sample for [^18^F]fluoro-[1,2-2H4]choline. A biodistribution study in mice with the HCT-116 tumors also confirmed the better in vivo stability and higher uptake of [^18^F]fluoro-[1,2-2H4]choline in different tissues, including tumor. Importantly, [^18^F]fluoro-[1,2-2H4]choline shows a considerably higher tumor uptake than [^18^F]fluorocholine at later time intervals.

### Stability of [O-methyl-^11^C]WAY-100635 vs [carbonyl-^11^C]WAY-100635

**Fig. 11:**
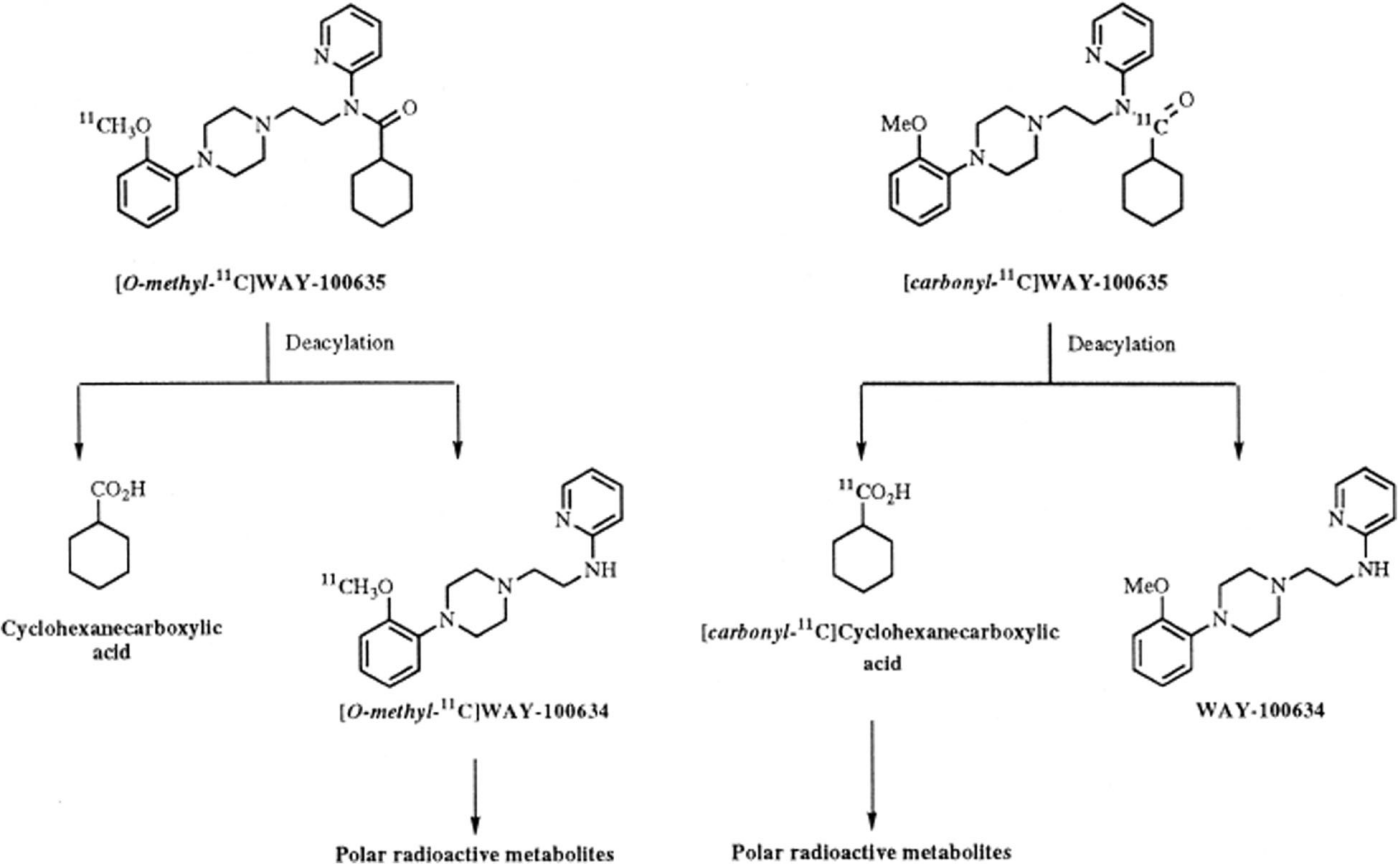
Pathways deduced for the metabolism of [O-methyl-^11^C]WAY-100635 and [carbonyl-^11^C]WAY-100635 in cynomolgus monkeys and humans. Adapted with permission from Ref. [23]

[Carbonyl-^11^C]WAY-100635 is one of the most promising PET radiotracers to assess 5-HT1A receptors in the brain. To study the influence of the label position on radiotracer characteristics, [carbonyl-11C]WAY-100635 and [O-methyl-11C]WAY-100635 were examined in a human and a healthy monkey by PET [23]. [Carbonyl-^11^C]WAY-100635 radioactivity was retained in regions including the occipital cortex, temporal cortex, and raphe nuclei, which are known to be affluent with 5-HT1A receptors. However, it rapidly cleared from the region that was almost devoid of 5-HT1A receptors such as the cerebellum. Compared to [O-methyl-^11^C]WAY-100635, [carbonyl-^11^C]WAY-100635 offers about 10 and 3 times superior signal contrast in receptor-affluent regions of the human and monkey brain, respectively. The HPLC analysis of plasma samples at various time periods helps detect the amount of parent radiotracers, [carbonyl-^11^C]WAY-100635, and its radiometabolites (Fig. 11) [23]. The original radiotracer constitutes 19% of the total radioactivity at 47 min in the monkey plasma, and in the human plasma, it constitutes around 8% of the overall radioactivity, after 40 min. In the human plasma, a negligible radiometabolite was detected as [carbonyl-^11^C]desmethyl-WAY-100635 but it was almost undetectable in the monkey plasma. [^11^C]Cyclohexanecarboxylicacid has been detected as the major radioactive metabolite, and it contributes to around 21% of the total radioactivity in the human plasma at 10 min after injection. In order to identify its effects in PET imaging, [carbonyl-^11^C]cyclohexanecarboxylic acid was administrated into the cynomolgus monkeys and the PET study was then performed. The PET examination concludes that the [^11^C]cyclohexanecarboxylicacid uptake was extremely low in the brain tissue and that it does not affect the PET study.

### Stability of [^18^F]FMeNER vs [^18^F]FMeNER-D2

(S,S)-[^11^C] MeNER displays high selective affinity to norepinephrine transporter (NET) [82]. However, there is an inherent problem of using (S,S)-[^11^C] MeNER in PET study of NET as the specific binding did not attain its maximum within the stipulated PET scanning period (93 min). Besides, the radiotracer also shows the significant noisy signal at later time point [82]. To avoid this problem, radiofluorinated analogs of (S,S)-[^11^C]MeNER were investigated. In 2004, Magnus et al. examined the PET study of NET using two fluorinated analog of (S,S)-[^11^C] MeNER—(S,S)-[18F]FMeNER and also (S,S)-[18F]FMeNER-D2 [83]. In the case of (*S,S*)-[^18^F]FMeNER, it was noticed that there was significant contamination in PET images due to the skull-bound radioactivity. This observation indicates the rapid defluorination of [^18^F]FMeNER. However, a significant decrease in defluorination occurs for the di-deuterated analog [^18^F]FMeNER-D2. These results prove that the defluorination rate of the radiotracer can be minimized by the deuterium isotope effect.

### Stability of [^18^F]FEPEP, [^18^F]FMPEP vs [^18^F]FMPEP-d2

^11^C-MePPEP, a PET radiotracer, has been used to quantify cannabinoid CB1 receptors in the brain [84]. However, the application of the^11^C-MePPEP was challenging due to the short half-life of the carbon-11 radioisotope (20.4 min). To overcome this limitation, ^18^F-labeled analogs of the MePPEP were developed (^18^F-FEPEP, ^18^F-FMPEP, and ^18F^-FMPEP-d2). Terry et al. examined the brain uptake of these three fluorinated tracers and identified that ^18^F-FMPEP-d2 is the best tracer due to its high brain uptake [85]. Besides, ^18^F-FMPEP-d2 displays a significantly lower radioactivity uptake in the skull compared to ^18^F-FMPEP. These results reveal that the accumulation of the 18F-fluoride ion in the bone occurs due to the in vivo defluorination of ^18^F-FMPEP.

### Stability of verapamil analog radiotracers

**Fig. 12:**
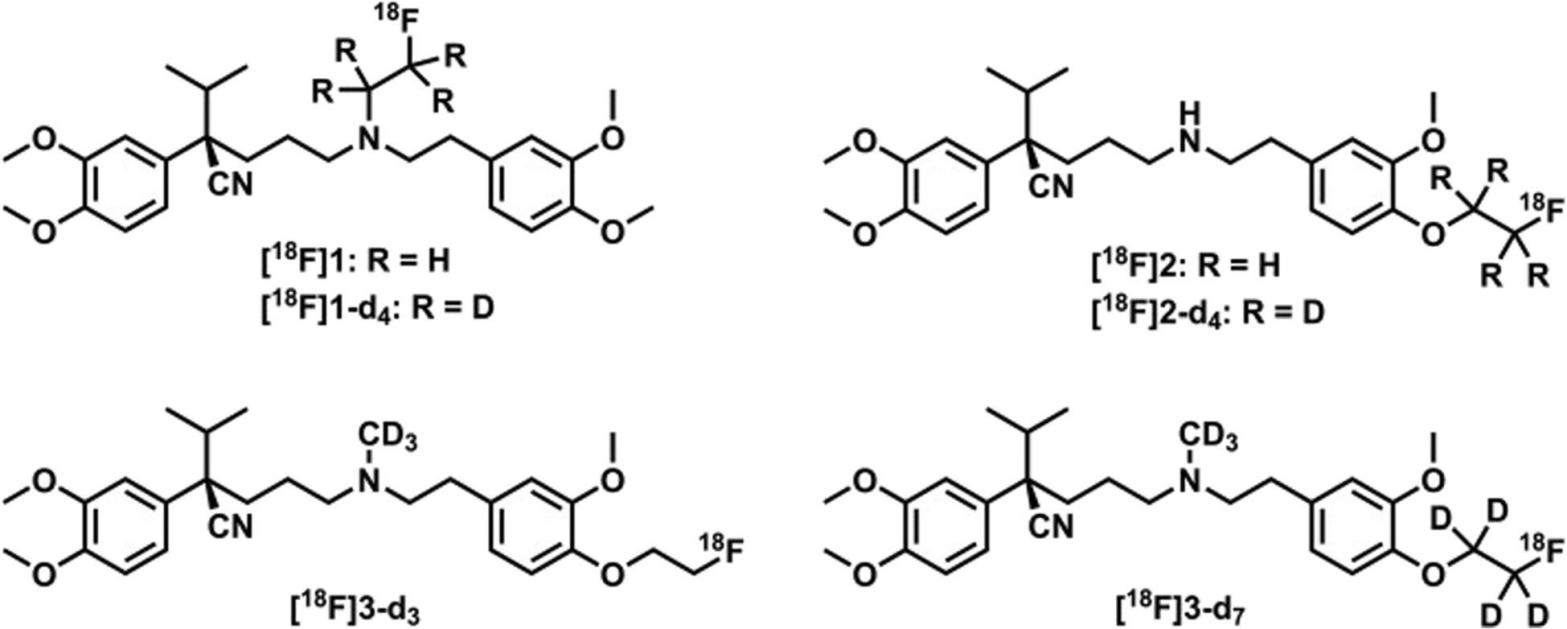
Chemical structures of deuterated (nor-)verapamil analogs. Adapted with permission from Ref. [87]

(R)-[^11^C] Verapamil is a widely used PET radiotracer for P-gp research. Two other analogs of verapamil, labeled with fluorine-18, were also reported [86] ([^18^F]1 and [^18^F]2, Fig. 12) to assess the P-gp function in the brain. However, all these three radiotracers suffer from complicated image interpretation due to their poor metabolic stability [86, 88]. Hence, to develop a stable radiotracer for P-gp, Raaphorst et al. have synthesized six new deuterium substituted analogs of verapamil and evaluated their metabolic stability [87]. They also aimed to evaluate their metabolic pathway. It was discovered that the metabolic stability of [^18^F]1 and [^18^F]2 could be enhanced by the addition of deuterium in the parent radiotracer. They also observed that the deuterium substituted methyl containing analogs ([^18^F]3-d3 and [^18^F]3-d7) showed better metabolic stability compared to their corresponding unsubstituted analogs. This increased metabolic stability could be due to enhanced steric hindrance which eventually slows down the enzymatic metabolism. In PET imaging, [^18^F]3-d7 and (R)-[^11^C] verapamil both have shown similar kind of in vivo characteristics. Therefore, [^18^F]3-d7 could be a potential metabolically stable radiotracer for the clinical evaluation of P-gp.

## Conclusion

PET is presently one of the most beneficial imaging tools for measuring drug pharmacokinetics non-invasively in the tissue of interest. Since the last decade, PET studies have offered a lot of important information regarding the tissue accumulation of numerous types of drugs. However, for the analysis and interpretation of the images, it is required to keep in mind that the collected signals from the PET scanner alone do not permit any conclusion to be drawn about the compound’s chemical identity that contributes to the radioactivity during the scan. The identification of radiometabolites is extremely important as they could offer some important information about the appropriate part of the compound to attach the radioactive label. From the image analysis point of view, detailed knowledge of the radiometabolite formation is required to get the maximum information from a PET scan. However, the identification of the radiometabolites is quite difficult because of its very short-lived radioisotope and the low dosage of the radiopharmaceutical and its metabolites. The tedious sample preparation procedure makes it even more complicated to analyze the radiometabolite in in vivo. Here, in this review, we have given important information and updates regarding the radiometabolite analysis of several carbon-11- and fluorine-18-based radiotracers from in vitro, preclinical and clinical aspects.

## Data Availability

Not applicable

## Abbreviations

CNS: Central nervous system
CT: Computed tomography
HPLC: High-performance liquid chromatography
HPLC-MS: High-performance liquid chromatography-mass spectrometry
MRI: Magnetic resonance imaging
OPTLC: Overpressure thin-layer chromatography
PET: Positron emission tomography
PPB: Plasma protein binding
SPE: Solid phase extraction
SERT: Serotonin transporter
TLC: Thin-layer chromatography
UHPLC/Q-ToF-MS: Ultra high-performance liquid chromatography/quadrupole-time of flight-mass spectrometry
